# Spontaneous Penile (Cavernosal) Abscess: Case Report with Discussion of Aetiology, Diagnosis, and Management with Review of Literature

**DOI:** 10.1100/tsw.2005.4

**Published:** 2005-01-21

**Authors:** Jayesh Sagar, Bethani Sagar, D. K. Shah

**Affiliations:** Stoke Mandeville Hospital, Aylesbury, Bucks, UK

**Keywords:** penile abscess, corpora cavernosa, surgical drainage, chordee

## Abstract

The rare presentation of spontaneous, corpus cavernosal abscess with evident pus discharge is reported. The 19-year-old English man was successfully treated with surgical drainage and antibiotics with long-term sequelae in form of mild, left-sided penile deviation, but normal erectile function. Though he did not require any further surgical intervention for correction of chordee at that time, there remains a possibility of it getting worse over time, which may ultimately need surgery for correction. The possible aetiology, diagnosis, and treatment of this rare condition are briefly discussed.

## INTRODUCTION

Abscess of the penis (corpus cavernosum) is an unusual condition, not frequently documented in literature. The most common pathogen is *Neisseria gonorrhea*, although other pathogens have been implicated. Few cases have been noticed following puncture of the corpus cavernosum for cavernosography or following injections for treatment of impotence. Here, this rare case of spontaneous penile abscess is presented, which was successfully treated by surgical drainage and antibiotics with long-term sequelae in form of mild, left-sided penile deviation, but normal erectile function. Though he did not require any surgical correction of chordee at that time, it may ultimately need surgical correction if it gets worse over time. The aetiology, diagnosis, and management of this rare condition are discussed briefly.

## CASE REPORT

A 19-year-old, fit and healthy English man working in the Royal Navy was referred to us by a general practitioner with complaints of gradual development of penile swelling over period of 6 days. Initially, it was small and painless, but became quite big and painful in the last 2 days of presentation. There was no history of pyrexia, trauma, scratching of genitalia, dysuria, urethral discharge, or history of gonorrhoea or dental caries. The general practitioner already started him on flucloxacilline, but he did not respond to it. He was heterosexual engaged with single sexual partner. He did not have sexual intercourse for last 2 months. He was circumcised about 6 years ago. He was not on any regular medications and he denied any history of diabetes, any other systemic disorders, any immunodeficiency disorder, skin infection, or sexually transmitted diseases.

On physical examination, he was apyrexial and haemodynamically normal. The local examination revealed an enlarged, swollen erythematous penis with tenderness, very much marked on the distal shaft of penis, predominantly on the right side than the left side. The most important feature was pus pointing with pus discharge on ventral aspect of the distal penis on the right side near corona glandis. The testicular and epididymal, digital rectal, and inguinal lymph node examinations did not reveal any abnormality. The systemic examination did not reveal any positive finding for any septic focus. Thus, the diagnosis of spontaneous penile abscess was made on clinical basis, as there was no apparent cause of his penile abscess on history and clinical examination. The laboratory studies showed normal white cell count (7700/mm cube) and CRP of 37.4. The rest of the blood investigations were normal. The urinalysis was normal as well. The blood culture was sent on admission. As the diagnosis was evident on clinical examination, further investigation in the form of ultrasonography was not performed. The decision for surgical drainage was taken after patient's consent as there was evident pus pointing with pus discharge. During surgical drainage, the tunica albuginea was opened through the pus-pointing site and about 7 ml of pus was drained from corpus cavernosum. There was a significant amount of inflammation in the surrounding area. After thorough cleansing, the incision was closed with absorbable sutures. The pus was sent for microscopy, culture, and sensitivity. Postoperatively, he was started on augmentine for 5 days. He recovered fairly well after drainage, having strong and uninterrupted urinary stream without any further recurrence of abscess. The blood culture did not reveal any pathogen even on 7 days incubation. The pus revealed Gram-positive, coagulase-negative *Staphylococcus aureus*. Assessment in outpatient in 4 weeks revealed presence of minimal induration and slight left-sided penile deviation (chordee) on erection, but without any interference in sexual activity and function.

## DISCUSSION

Penile abscess is an uncommon clinical entity. It occurs more commonly as complication of gonorrhoea with or without associated urethritis [1] *N. gonorrhoea* has been found in prepuce, glans penis and most commonly in median raphe of penis [2], though the other pathogens have been implicated as well[3,4]. However corporal infection and abscess formation have been reported with priapism[5], cavernosography, intracavernosal injection, trauma [6] and foreign body. It is yet not clear that corpora cavernosa are resistant to haematogenous spread of infection. There has been report of a case where dental caries have been blamed as a cause of penile abscess through haematogenous dissemination[7]. In this presented patient, we could not find any causative factor for penile abscess formation, hence titled as spontaneous penile abscess. The other interesting point about this case is its bilateral presentation[8].

The spontaneous development of cavernosal abscess with pus pointing and discharge has been reported very rarely in literature. There have been very few cases documented in literature reporting coagulase negative staphylococcus aureus as responsible micororganism for cavernosal abscess formation[9]. Though it is frequently found in micro flora, it can be a significant opportunistic infective pathogen under certain conditions. But in our case we admit that out patient was a fit and healthy man without any significant medical conditions[10], so possibility of staphylococcus aureus as contaminant cannot be denied.

In the past, the diagnosis of penile abscess was mainly dependent on clinical examination and aspiration. But with the advent of technology, clinical doubt about the diagnosis can be confirmed by ultrasonography and at the same time it can allow the opportunity to aspirate the contents under ultrasonographic control, and thus works as therapeutic measure as well[11]. In our patient, we did not use radiological help, as the diagnosis was evident on clinical examination with pus pointing and pus discharge. Prompt drainage of pus form cavernosal bodies is the prime aim of treatment of this condition. This can be performed under ultrasonographic control or can be achieved by formal surgical drainage. We preferred open surgical drainage in our case due to evident pus pointing and pus discharge. Usually the recovery is well with help of postoperative antibiotics. The common possible complications include poor erectile function affecting sexual activity and secondary fibrosis leading to penile deviation[12]. These complications are more common with open surgical drainage that ultrasonic aspiration of penile abscess. In our case, patient developed slight left sided penile deviation following open surgical drainage though it did not affect his erectile function and sexual life. At that time our patient did not require surgery for chordee but he may ultimately need surgical correction of chordee if it gets worse over time. One could argue that if we would have chosen ultrasonic aspiration of penile abscess, we could have avoided or at least lessen the postoperative chordee. These postoperative complications can be managed, if required, by implantation of penile prosthesis or plastic surgical intervention to correct the penile deviation.

## References

1. Yamada, Y. and Ito, K. (2001) Gonococcal abscess in the prepuce: a case report. *Kansenshogaku Zasshi.* **75(9)**:819-821.
2. Clifford, G.R,, Krieger, J.N., and Rein, M.F. Gonococcal infection of the median raphe. (1983) *J Urology*. **130(1)**, 138-139.
3. Neidrach, W.L., Lerner, R.M., and Linke, C.A. Penile abscess involving corpus cavernosum: A case report. (1989) *The Journal of Urology*. **141(2),** 374-375.
4. Fernandez Duran, A.M., Martin, Garcia C., Fernandez Gomez, J., Jime Lopez-Lucendo, N., and Sampietro Crespo, A. Priapism secondary to a bilateral abscess of the corpora cavernosa. (1999) *Actas Urol Esp*. **23(1),** 64-66.
5. Herrera Puerto, J., Herrera Flores, J., Fernandez Iflesias, J., Marreno Calvo, M., and Burguillo Jimenez, N. (2003) Unusual complication post circumcision. *Actas Urol. Esp.* **27(7)**, 559–561.
6. Sater AA, Vandendris M. Abscess of Corpus Cavernosum. (1989) *The Journal of Urology*. **141(4)**, 949.
7. Fernandez Gomez JM, Regadera Sejas FJ, Perez Garcia FJ, Sahagu Arguello JL. Bilateral abscess of cavernous bodies. (1997) *Actas Urol Esp*. **21(1)**, 75-77.
8. Koksai T, Kadioglu A, Tefekli A, Usta M, Besisik A, Erol B. Spontaneous bacterial abscess of bilateral cavernosal bodies. (1999) *BJU International*. **84(9),** 1107-1108.
9. Moskovitz B, Vardi Y, Pery M, Bolkier M, Levin DR. Abscess of corpus cavernosum. (1992). *Urol Int*. **48(4),** 439-440.
10. Kropman RF, de la Fuente RB, Venema PL, van Imhoff WL. Treatment of corpus cavernosum abscess by aspiration and intravenous antibiotics. (1993). *J Urology.* **150(5 Pt 1),** 1502-1503.
11. Pascual Regueiro D, Garcia de Jalon Martinez A, Mallen Mateo E, Sancho Serrano C, Borque Fernando A, Rioja Sanz LA. Penile curvature secondary to cavernous body abscess.(2003). *Actas Urol Esp*. **27(1),** 55-59.
12. Kameda, K., Hayashi, N., Arima, K., Yanagawa, M., Kawamaru, J., Yone, S., and Kinbara, H. Abscess of corpus cavernosum: a case report: *Hinyokika Kiyo* (1998). **44(12),** 893-895.
